# Complete Genome Sequence of Nonagglutinating *Lactococcus garvieae* Strain 122061 Isolated from Yellowtail in Japan

**DOI:** 10.1128/genomeA.00592-16

**Published:** 2016-07-07

**Authors:** Issei Nishiki, Daisaku Oinaka, Yuki Iwasaki, Motoshige Yasuike, Yoji Nakamura, Terutoyo Yoshida, Atushi Fujiwara, Satoshi Nagai, Masaya Katoh, Takanori Kobayashi

**Affiliations:** a Research Center for Bioinformatics and Biosciences, National Research Institute of Fisheries Science, Japan Fisheries Research and Education Agency, Yokohama, Japan; b Faculty of Agriculture, University of Miyazaki, Miyazaki, Japan

## Abstract

Nonagglutinating *Lactococcus garvieae* has been isolated from diseased farmed yellowtail in Japan since 2012. In this study, the complete genome and plasmid sequence of nonagglutinating *L. garvieae* strain 122061 was determined, to our knowledge, for the first time.

## GENOME ANNOUNCEMENT

*Lactococcus garvieae* is a Gram-positive, α-hemolytic, nonmotile, and non-spore-forming ovoid coccus that forms short chains and white colonies. In aquaculture, yellowtail infected with *L. garvieae* show high mortality (1–3). A protective vaccine containing formalin-inactivated cells, which can be injected or orally administered, was developed for the genus *Seriola*, dramatically decreasing the damage caused by *L. garvieae* infection (4, 5). In Japan, a slide agglutination test with rabbit antiserum is routinely used to identify *L. garvieae* isolated from fish (6).

Since 2012, *L. garvieae*, which does not agglutinate with regular antiserum, has been isolated from vaccinated yellowtail (*Seriola quinqueradiata*). The isolates were identified as nonagglutinating *L. garvieae* (7). The biochemical properties and morphological characteristics of nonagglutinating *L. garvieae* were the same as those of typical *L. garvieae* (7). However, nonagglutinating *L. garvieae* differed from typical *L. garvieae* in terms of the susceptibility to lytic phage and antigenicity of autoclave-extracted cellular antigens (7). Interestingly, the protective effect of a commercially available vaccine against nonagglutinating *L. garvieae* was found to be limited (8). Thus, nonagglutinating *L. garvieae* is one of the most dangerous pathogens of fish farms in Japan. To comprehensively analyze the genome of nonagglutinating *L. garvieae*, we determined the complete genome sequence of nonagglutinating *L. garvieae* strain 122061.

*L. garvieae* strain 122061 was isolated from vaccinated yellowtail in 2012 and grown in Todd Hewitt Broth (Difco, Sparks, MD, USA) at 25°C for 24 h. Genomic DNA was extracted using the Maxwell Cell DNA purification kit (Promega, Madison, WI, USA) and used to construct SMRTbell sequencing libraries (Pacific Biosciences, Menlo Park, CA, USA). The whole-genome sequence was determined using PacBio RS II (Pacific Biosciences). *De novo* assembly using Hierarchical Genome Assembly Process 3 (PacBio DevNet; Pacific Biosciences) generated two circular contigs composed of a chromosome and a plasmid. Functional annotation was performed using Prokka (http://www.vicbioinformatics.com/software.prokka.shtml). The chromosomal genome was 1,985,694 bp with a G+C content of 38.2%. The plasmid was 14,414 bp with a G+C content of 34.7%. Prokka predicted a total of 2,036 genes including 1,961 coding genes, 16 rRNA genes, and 59 tRNA genes in the chromosomal genome of nonagglutinating *L. garvieae* strain 122061. The plasmid was predicted to contain 16 coding genes. The average nucleotide identity (ANI) between the typical *L. garvieae* strain lg2 (AP090129) and nonagglutinating *L. garvieae* strain 122061 was estimated at 90.7% by the ANI calculator (http://enve-omics.ce.gatech.edu/ani/index). Here, we report the complete genome sequence of nonagglutinating *L. garvieae* for, to our knowledge, the first time. This genome sequence can be used to comparatively analyze the genomes of different *L. garvieae* strains.

### Nucleotide sequence accession numbers.

The complete annotated genome and plasmid sequences of nonagglutinating *L. garvieae* strain 122061 were deposited in GenBank under the accession numbers AP017373 and AP017399, respectively.
